# Crystal structure of 1-[(2,4,6-triiso­propyl­phen­yl)sulfon­yl]aziridine

**DOI:** 10.1107/S2056989015010221

**Published:** 2015-06-03

**Authors:** Lena Knauer, Christopher Golz, Carsten Strohmann

**Affiliations:** a Fakultät für Chemie und Chemische Biologie, Technische Universität Dortmund, Otto-Hahn-Strasse 6, 44227 Dortmund, Germany

**Keywords:** crystal structure, aziridine, triiso­propyl­benzene­sulfon­yl, consecutive ring-opening reactions

## Abstract

The title compound, C_17_H_27_NO_2_S, exhibits a distorted geometry of the aromatic ring with elongated bonds at the *ipso*-C atom. The S atom deviates from the aromatic ring plane by 0.393 (4) Å. Similar to this, the adjacent isopropyl groups are bent out of the aromatic ring plane by −0.125 (4) and −0.154 (4) Å. Even the distant isopropyl group in *para*-position to the sulfonyl moiety shows a slight deviation from the ring plane of 0.111 (5) Å. These distortions, which are caused by the bulky substituents, can also be observed in related sulfonyl­aziridine structures.

## Related literature   

For the crystal structure of a related phenyl-substituted compound, see: Golz *et al.* (2014 ▸). For a discussion of the geometry of the triiso­propyl­benzene­sulfonyl moiety, see: Sandrock *et al.* (2004 ▸). For a discussion of the pyramidalized geometry of *N*-sulfonyl­amides, see: Ohwada *et al.* (1998 ▸). By regioselective ring opening reactions, countless nitro­gen-containing compounds are accessible, see: Stamm (1999 ▸); Schneider (2009 ▸). For consecutive ring-opening reactions of aziridines by tri­ethyl­amine, see: Golz & Strohmann (2015 ▸). In some cases, the three-membered aziridine ring is further activated by electron-withdrawing groups (Hu, 2004 ▸) to increase its reactivity.
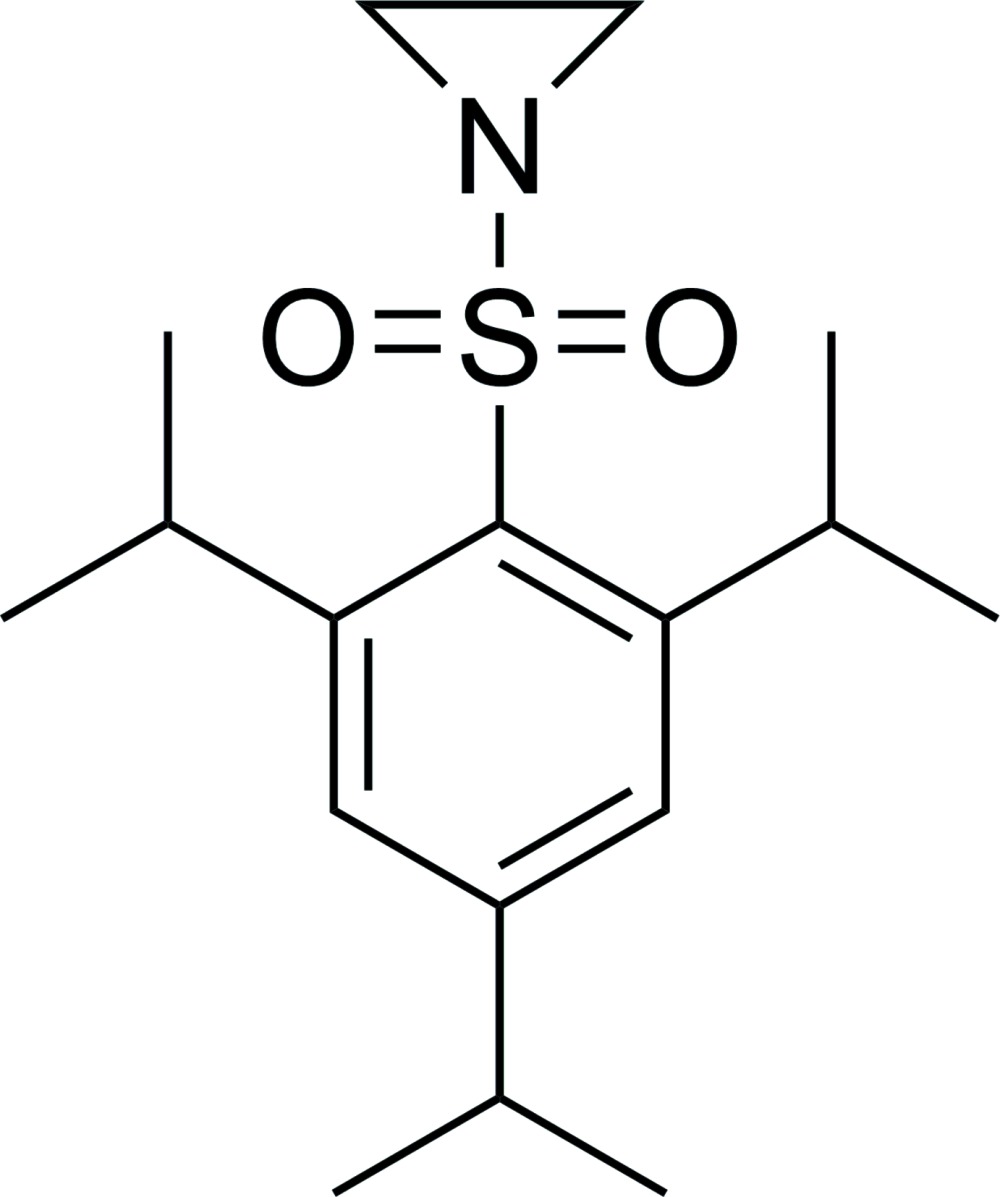


## Experimental   

### Crystal data  

C_17_H_27_NO_2_S
*M* *_r_* = 309.45
Monoclinic, 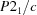

*a* = 6.2679 (8) Å
*b* = 17.5289 (18) Å
*c* = 16.3890 (13) Å
β = 100.331 (10)°
*V* = 1771.5 (3) Å^3^
*Z* = 4
Mo *K*α radiation
μ = 0.19 mm^−1^
*T* = 173 K
0.33 × 0.25 × 0.01 mm

### Data collection  

Agilent Xcalibur Sapphire3 diffractometer
Absorption correction: multi-scan (*CrysAlis PRO*; Agilent, 2013 ▸) *T*_min_ = 0.782, *T*_max_ = 1.000
9521 measured reflections
3449 independent reflections
2479 reflections with *I* > 2σ(*I*)
*R*_int_ = 0.049

### Refinement  

*R*[*F* ^2^ > 2σ(*F* ^2^)] = 0.053
*wR*(*F* ^2^) = 0.141
*S* = 1.07
3449 reflections
196 parameters
H-atom parameters constrained
Δρ_max_ = 0.33 e Å^−3^
Δρ_min_ = −0.58 e Å^−3^

### 

Data collection: *CrysAlis PRO* (Agilent, 2013 ▸); cell refinement: *CrysAlis PRO*; data reduction: *CrysAlis PRO*; program(s) used to solve structure: *SHELXS97* (Sheldrick, 2008 ▸); program(s) used to refine structure: *SHELXL2014* (Sheldrick, 2015 ▸); molecular graphics: *OLEX2* (Dolomanov *et al.*, 2009 ▸); software used to prepare material for publication: *OLEX2*.

## Supplementary Material

Crystal structure: contains datablock(s) I. DOI: 10.1107/S2056989015010221/fk2088sup1.cif

Structure factors: contains datablock(s) I. DOI: 10.1107/S2056989015010221/fk2088Isup2.hkl

Supporting information file. DOI: 10.1107/S2056989015010221/fk2088Isup3.cml

**Fig. 1.**
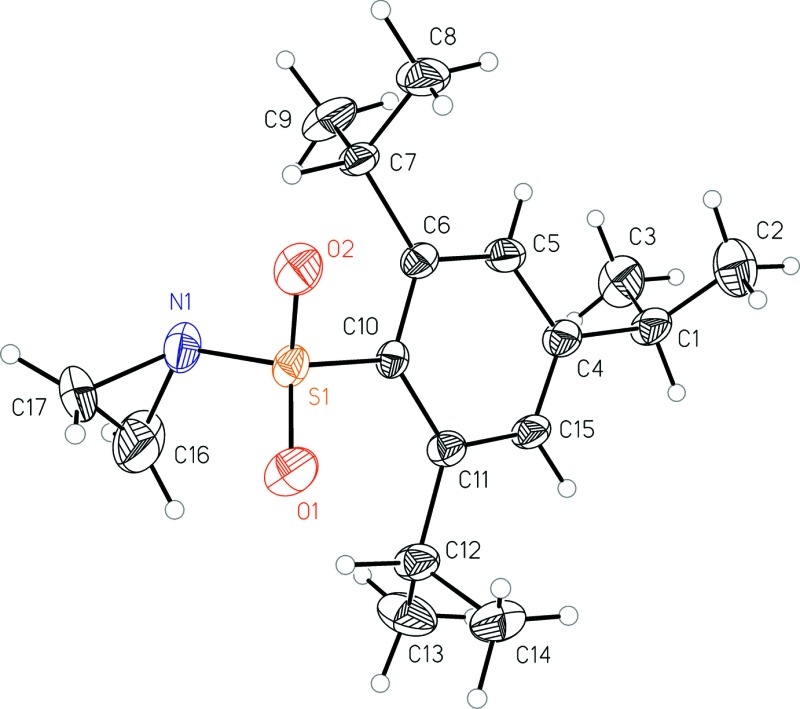
Molecular structure of the title compound with anisotropic displacement ellipsoids drawn at 50% probability level.

. DOI: 10.1107/S2056989015010221/fk2088fig1.tif
Mol­ecular structure of the title compound with anisotropic displacement ellipsoids drawn at 50% probability level.

CCDC reference: 1403377

Additional supporting information:  crystallographic information; 3D view; checkCIF report

## supplementary crystallographic information

### S1. Structural commentary

Aziridines are inter­esting and versatile building blocks in synthetic chemistry due to their high ring strain. By regioselective ring opening reactions, countless nitro­gen-containing compounds are accessible (Stamm, 1999; Schneider, 2009). For example, the aziridine ring can be opened by tri­ethyl­amine (Golz & Strohmann, 2015). In some cases, the three-membered aziridine ring is further activated by electron-withdrawing groups (Hu, 2004) to increase its reactivity.

The title compound, C_17_H_27_NO_2_S, is a representative of the class of activated aziridines, as it contains a triiso­propyl­benzene substituted sulfonyl ester attached to the nitro­gen atom. In the aromatic ring, the bulky substituents lead to a distortion of its geometry. This is expressed by the increased bond lengths and out-of-plane bent substituents around the benzene ring. At the *ipso*-carbon, the bonds C10–C11 and C10–C6 are slightly elongated to 1.410 (3) Å. In contrast, the other bonds of the aromatic ring exhibit usual lengths [C4–C15 1.374 (3) Å, C5–C61.380 (3) Å, C11–C15 1.388 (3) Å, C4–C5 1.389 (3) Å]. The sulfonyl group as well as the adjacent iso­propyl groups bend out of the aromatic plane. This is caused by steric repulsion between the iso­propyl groups and the sulfonyl oxygen atoms. The sulfur atom deviates from the mean aromatic ring plane by 0.393 (4) Å. The carbon atoms C7 and C12 show distances of -0.125 (4) Å and -0.154 (4) Å, respectively (see Table 5). A similar distortion can also be observed at the iso­propyl group in *para* position to the sulfonyl moiety. Here, C1 has a distance to the aromatic ring plane of 0.111 (5) Å, thus being distorted in the same direction as the sulfur atom. This is caused by steric repulsion between the C1 iso­propyl group and the adjacent iso­propyl groups in *ortho* position in respect of the sulfonyl moiety.

### S2. Refinement

Crystal data, data collection and structure refinement details are summarized in Table 1.

Hydrogen atoms were located from difference Fourier maps, refined at idealized positions riding on the carbon atoms with isotropic displacement parameters U_iso_(H) = 1.2U_eq_(C) or 1.5U_eq_(–CH_3_) and aromatic C–H = 0.95 Å, primary C–H = 0.98 Å, secondary C–H = 0.99 Å, tertiary C–H = 1.00 Å. All CH_3_ hydrogen atoms were allowed to rotate but not to tip. Aziridine protons could be located from difference Fourier maps, but were refined as idealized CH_2_ groups.

### Crystal data

**Table:**

C_17_H_27_NO_2_S	*F*(000) = 672
*M**_r_* = 309.45	*D*_x_ = 1.160 Mg m^−^^3^
Monoclinic, *P*2_1_/*c*	Mo *K*α radiation, λ = 0.71073 Å
*a* = 6.2679 (8) Å	Cell parameters from 2277 reflections
*b* = 17.5289 (18) Å	θ = 3.3–29.2°
*c* = 16.3890 (13) Å	µ = 0.19 mm^−^^1^
β = 100.331 (10)°	*T* = 173 K
*V* = 1771.5 (3) Å^3^	Plate, colourless
*Z* = 4	0.33 × 0.25 × 0.01 mm

### Data collection

**Table:**

Agilent Xcalibur Sapphire3 diffractometer	3449 independent reflections
Radiation source: Enhance (Mo) X-ray Source	2479 reflections with *I* > 2σ(*I*)
Graphite monochromator	*R*_int_ = 0.049
Detector resolution: 16.0560 pixels mm^-1^	θ_max_ = 26.0°, θ_min_ = 2.5°
ω scans	*h* = −7→7
Absorption correction: multi-scan (*CrysAlis PRO*; Agilent, 2013)	*k* = −15→21
*T*_min_ = 0.782, *T*_max_ = 1.000	*l* = −20→20
9521 measured reflections	

### Refinement

**Table:**

Refinement on *F*^2^	0 restraints
Least-squares matrix: full	Hydrogen site location: inferred from neighbouring sites
*R*[*F*^2^ > 2σ(*F*^2^)] = 0.053	H-atom parameters constrained
*wR*(*F*^2^) = 0.141	*w* = 1/[σ^2^(*F*_o_^2^) + (0.0473*P*)^2^ + 0.8936*P*] where *P* = (*F*_o_^2^ + 2*F*_c_^2^)/3
*S* = 1.07	(Δ/σ)_max_ < 0.001
3449 reflections	Δρ_max_ = 0.33 e Å^−^^3^
196 parameters	Δρ_min_ = −0.58 e Å^−^^3^

### Special details

**Table:**

Experimental. Absorption correction: CrysAlisPro, Agilent Technologies, Version 1.171.36.28 (release 01-02-2013 CrysAlis171 .NET) (compiled Feb 1 2013,16:14:44) Empirical absorption correction using spherical harmonics, implemented in SCALE3 ABSPACK scaling algorithm.
Geometry. All e.s.d.'s (except the e.s.d. in the dihedral angle between two l.s. planes) are estimated using the full covariance matrix. The cell e.s.d.'s are taken into account individually in the estimation of e.s.d.'s in distances, angles and torsion angles; correlations between e.s.d.'s in cell parameters are only used when they are defined by crystal symmetry. An approximate (isotropic) treatment of cell e.s.d.'s is used for estimating e.s.d.'s involving l.s. planes.

### Fractional atomic coordinates and isotropic or equivalent isotropic displacement parameters (Å^2^)

**Table:**

	*x*	*y*	*z*	*U*_iso_*/*U*_eq_	
S1	0.71473 (10)	0.20619 (4)	0.37952 (4)	0.0245 (2)	
C10	0.7527 (4)	0.26810 (13)	0.29748 (14)	0.0205 (5)	
C15	0.8227 (4)	0.28863 (14)	0.16069 (15)	0.0272 (6)	
H15	0.8229	0.2705	0.1061	0.033*	
C6	0.8105 (4)	0.34434 (14)	0.31802 (14)	0.0246 (6)	
C5	0.8842 (4)	0.38836 (15)	0.25903 (15)	0.0297 (6)	
H5	0.9273	0.4394	0.2726	0.036*	
C11	0.7462 (4)	0.24050 (14)	0.21624 (15)	0.0243 (6)	
C4	0.8981 (4)	0.36110 (15)	0.18058 (15)	0.0279 (6)	
O1	0.7219 (3)	0.12858 (10)	0.35547 (11)	0.0370 (5)	
O2	0.8625 (3)	0.22687 (11)	0.45315 (10)	0.0360 (5)	
N1	0.4729 (4)	0.23217 (13)	0.39562 (14)	0.0345 (6)	
C17	0.3491 (5)	0.17365 (19)	0.4317 (2)	0.0500 (9)	
H17A	0.4185	0.1234	0.4451	0.060*	
H17B	0.2536	0.1908	0.4702	0.060*	
C16	0.2886 (6)	0.1945 (3)	0.3447 (2)	0.0701 (12)	
H16A	0.1543	0.2246	0.3280	0.084*	
H16B	0.3190	0.1572	0.3029	0.084*	
C12	0.6577 (5)	0.16388 (14)	0.18281 (15)	0.0312 (6)	
H12	0.5872	0.1391	0.2261	0.037*	
C7	0.7975 (4)	0.38301 (14)	0.40041 (15)	0.0290 (6)	
H7	0.7291	0.3463	0.4347	0.035*	
C1	0.9900 (5)	0.41027 (15)	0.11913 (15)	0.0331 (7)	
H1	0.9766	0.3811	0.0660	0.040*	
C13	0.4860 (6)	0.17400 (18)	0.10515 (18)	0.0489 (9)	
H13A	0.3751	0.2099	0.1166	0.073*	
H13B	0.4184	0.1246	0.0887	0.073*	
H13C	0.5539	0.1940	0.0602	0.073*	
C3	0.8614 (5)	0.48402 (17)	0.10107 (18)	0.0457 (8)	
H3A	0.7095	0.4719	0.0787	0.068*	
H3B	0.9223	0.5142	0.0605	0.068*	
H3C	0.8695	0.5133	0.1525	0.068*	
C14	0.8399 (5)	0.11176 (17)	0.1661 (2)	0.0471 (8)	
H14A	0.9167	0.1361	0.1259	0.071*	
H14B	0.7787	0.0631	0.1436	0.071*	
H14C	0.9415	0.1026	0.2180	0.071*	
C2	1.2285 (5)	0.42692 (19)	0.1493 (2)	0.0496 (9)	
H2A	1.2459	0.4567	0.2006	0.074*	
H2B	1.2856	0.4560	0.1068	0.074*	
H2C	1.3083	0.3788	0.1597	0.074*	
C8	1.0244 (5)	0.40142 (17)	0.44750 (16)	0.0407 (8)	
H8A	1.1099	0.3544	0.4564	0.061*	
H8B	1.0137	0.4243	0.5012	0.061*	
H8C	1.0951	0.4374	0.4151	0.061*	
C9	0.6542 (6)	0.45350 (17)	0.38647 (18)	0.0476 (8)	
H9A	0.7228	0.4919	0.3562	0.071*	
H9B	0.6351	0.4744	0.4401	0.071*	
H9C	0.5125	0.4396	0.3541	0.071*	

### Atomic displacement parameters (Å^2^)

**Table:**

	*U*^11^	*U*^22^	*U*^33^	*U*^12^	*U*^13^	*U*^23^
S1	0.0289 (4)	0.0256 (4)	0.0205 (3)	0.0015 (3)	0.0085 (3)	0.0032 (3)
C10	0.0213 (13)	0.0238 (13)	0.0168 (11)	0.0008 (10)	0.0048 (10)	0.0025 (10)
C15	0.0372 (16)	0.0275 (14)	0.0178 (12)	−0.0032 (12)	0.0077 (11)	−0.0036 (10)
C6	0.0284 (14)	0.0279 (14)	0.0174 (12)	−0.0012 (11)	0.0039 (11)	−0.0011 (10)
C5	0.0401 (17)	0.0269 (14)	0.0228 (13)	−0.0082 (12)	0.0077 (12)	−0.0035 (11)
C11	0.0283 (14)	0.0233 (13)	0.0212 (12)	0.0003 (11)	0.0043 (11)	−0.0005 (10)
C4	0.0358 (16)	0.0297 (15)	0.0190 (12)	−0.0034 (12)	0.0068 (11)	0.0003 (11)
O1	0.0590 (14)	0.0250 (10)	0.0292 (10)	0.0063 (9)	0.0137 (10)	0.0051 (8)
O2	0.0431 (13)	0.0460 (12)	0.0184 (9)	−0.0037 (9)	0.0036 (9)	0.0068 (8)
N1	0.0286 (13)	0.0448 (14)	0.0326 (12)	−0.0001 (11)	0.0128 (11)	0.0007 (11)
C17	0.0423 (19)	0.053 (2)	0.063 (2)	−0.0197 (15)	0.0294 (17)	−0.0063 (17)
C16	0.0294 (19)	0.123 (4)	0.059 (2)	−0.014 (2)	0.0122 (17)	−0.030 (2)
C12	0.0473 (18)	0.0251 (14)	0.0221 (13)	−0.0042 (12)	0.0088 (12)	−0.0015 (11)
C7	0.0442 (17)	0.0251 (14)	0.0197 (12)	−0.0084 (12)	0.0115 (12)	−0.0048 (11)
C1	0.0504 (18)	0.0296 (15)	0.0224 (13)	−0.0063 (13)	0.0152 (13)	−0.0008 (11)
C13	0.060 (2)	0.0442 (19)	0.0360 (16)	−0.0173 (16)	−0.0084 (16)	−0.0013 (14)
C3	0.060 (2)	0.0423 (19)	0.0371 (16)	−0.0001 (16)	0.0160 (16)	0.0155 (14)
C14	0.059 (2)	0.0336 (17)	0.0517 (19)	−0.0011 (15)	0.0181 (17)	−0.0145 (15)
C2	0.047 (2)	0.055 (2)	0.0497 (19)	−0.0071 (16)	0.0182 (16)	0.0124 (16)
C8	0.056 (2)	0.0432 (18)	0.0215 (13)	−0.0145 (15)	0.0028 (14)	−0.0060 (13)
C9	0.070 (2)	0.0402 (18)	0.0353 (16)	0.0090 (16)	0.0172 (16)	−0.0109 (14)

### Geometric parameters (Å, º)

**Table:**

S1—C10	1.777 (2)	C7—H7	1.0000
S1—O1	1.4193 (19)	C7—C8	1.526 (4)
S1—O2	1.4300 (19)	C7—C9	1.520 (4)
S1—N1	1.649 (2)	C1—H1	1.0000
C10—C6	1.410 (3)	C1—C3	1.524 (4)
C10—C11	1.410 (3)	C1—C2	1.517 (4)
C15—H15	0.9500	C13—H13A	0.9800
C15—C11	1.388 (3)	C13—H13B	0.9800
C15—C4	1.374 (4)	C13—H13C	0.9800
C6—C5	1.380 (3)	C3—H3A	0.9800
C6—C7	1.526 (3)	C3—H3B	0.9800
C5—H5	0.9500	C3—H3C	0.9800
C5—C4	1.389 (3)	C14—H14A	0.9800
C11—C12	1.518 (4)	C14—H14B	0.9800
C4—C1	1.516 (3)	C14—H14C	0.9800
N1—C17	1.473 (3)	C2—H2A	0.9800
N1—C16	1.456 (4)	C2—H2B	0.9800
C17—H17A	0.9900	C2—H2C	0.9800
C17—H17B	0.9900	C8—H8A	0.9800
C17—C16	1.455 (5)	C8—H8B	0.9800
C16—H16A	0.9900	C8—H8C	0.9800
C16—H16B	0.9900	C9—H9A	0.9800
C12—H12	1.0000	C9—H9B	0.9800
C12—C13	1.523 (4)	C9—H9C	0.9800
C12—C14	1.526 (4)		
			
O1—S1—C10	111.10 (11)	C8—C7—H7	107.9
O1—S1—O2	115.43 (12)	C9—C7—C6	110.6 (2)
O1—S1—N1	112.65 (12)	C9—C7—H7	107.9
O2—S1—C10	109.23 (11)	C9—C7—C8	112.1 (2)
O2—S1—N1	105.67 (12)	C4—C1—H1	107.8
N1—S1—C10	101.77 (11)	C4—C1—C3	111.1 (2)
C6—C10—S1	117.51 (17)	C4—C1—C2	111.3 (2)
C6—C10—C11	120.9 (2)	C3—C1—H1	107.8
C11—C10—S1	121.32 (19)	C2—C1—H1	107.8
C11—C15—H15	118.3	C2—C1—C3	110.9 (2)
C4—C15—H15	118.3	C12—C13—H13A	109.5
C4—C15—C11	123.4 (2)	C12—C13—H13B	109.5
C10—C6—C7	125.5 (2)	C12—C13—H13C	109.5
C5—C6—C10	117.8 (2)	H13A—C13—H13B	109.5
C5—C6—C7	116.7 (2)	H13A—C13—H13C	109.5
C6—C5—H5	118.6	H13B—C13—H13C	109.5
C6—C5—C4	122.7 (2)	C1—C3—H3A	109.5
C4—C5—H5	118.6	C1—C3—H3B	109.5
C10—C11—C12	126.2 (2)	C1—C3—H3C	109.5
C15—C11—C10	117.1 (2)	H3A—C3—H3B	109.5
C15—C11—C12	116.6 (2)	H3A—C3—H3C	109.5
C15—C4—C5	117.5 (2)	H3B—C3—H3C	109.5
C15—C4—C1	121.6 (2)	C12—C14—H14A	109.5
C5—C4—C1	120.9 (2)	C12—C14—H14B	109.5
C17—N1—S1	116.0 (2)	C12—C14—H14C	109.5
C16—N1—S1	116.1 (2)	H14A—C14—H14B	109.5
C16—N1—C17	59.5 (2)	H14A—C14—H14C	109.5
N1—C17—H17A	117.8	H14B—C14—H14C	109.5
N1—C17—H17B	117.8	C1—C2—H2A	109.5
H17A—C17—H17B	114.9	C1—C2—H2B	109.5
C16—C17—N1	59.7 (2)	C1—C2—H2C	109.5
C16—C17—H17A	117.8	H2A—C2—H2B	109.5
C16—C17—H17B	117.8	H2A—C2—H2C	109.5
N1—C16—H16A	117.7	H2B—C2—H2C	109.5
N1—C16—H16B	117.7	C7—C8—H8A	109.5
C17—C16—N1	60.8 (2)	C7—C8—H8B	109.5
C17—C16—H16A	117.7	C7—C8—H8C	109.5
C17—C16—H16B	117.7	H8A—C8—H8B	109.5
H16A—C16—H16B	114.8	H8A—C8—H8C	109.5
C11—C12—H12	107.9	H8B—C8—H8C	109.5
C11—C12—C13	110.9 (2)	C7—C9—H9A	109.5
C11—C12—C14	111.0 (2)	C7—C9—H9B	109.5
C13—C12—H12	107.9	C7—C9—H9C	109.5
C13—C12—C14	111.1 (2)	H9A—C9—H9B	109.5
C14—C12—H12	107.9	H9A—C9—H9C	109.5
C6—C7—H7	107.9	H9B—C9—H9C	109.5
C8—C7—C6	110.4 (2)		
			
S1—C10—C6—C5	166.7 (2)	C5—C6—C7—C8	−67.6 (3)
S1—C10—C6—C7	−13.6 (3)	C5—C6—C7—C9	57.1 (3)
S1—C10—C11—C15	−166.5 (2)	C5—C4—C1—C3	−58.7 (4)
S1—C10—C11—C12	15.3 (4)	C5—C4—C1—C2	65.4 (3)
S1—N1—C17—C16	−106.4 (3)	C11—C10—C6—C5	−7.3 (4)
S1—N1—C16—C17	106.1 (2)	C11—C10—C6—C7	172.4 (3)
C10—S1—N1—C17	154.1 (2)	C11—C15—C4—C5	−3.9 (4)
C10—S1—N1—C16	87.0 (2)	C11—C15—C4—C1	177.0 (3)
C10—C6—C5—C4	1.6 (4)	C4—C15—C11—C10	−1.5 (4)
C10—C6—C7—C8	112.8 (3)	C4—C15—C11—C12	176.8 (2)
C10—C6—C7—C9	−122.6 (3)	O1—S1—C10—C6	−162.51 (19)
C10—C11—C12—C13	125.2 (3)	O1—S1—C10—C11	11.4 (3)
C10—C11—C12—C14	−110.8 (3)	O1—S1—N1—C17	35.1 (2)
C15—C11—C12—C13	−52.9 (3)	O1—S1—N1—C16	−32.0 (3)
C15—C11—C12—C14	71.0 (3)	O2—S1—C10—C6	−34.0 (2)
C15—C4—C1—C3	120.4 (3)	O2—S1—C10—C11	139.9 (2)
C15—C4—C1—C2	−115.5 (3)	O2—S1—N1—C17	−91.8 (2)
C6—C10—C11—C15	7.2 (4)	O2—S1—N1—C16	−158.9 (2)
C6—C10—C11—C12	−170.9 (2)	N1—S1—C10—C6	77.4 (2)
C6—C5—C4—C15	3.8 (4)	N1—S1—C10—C11	−108.7 (2)
C6—C5—C4—C1	−177.1 (3)	C7—C6—C5—C4	−178.1 (3)
