# Supplementary figures and images for: Crystal structure of 1-[(2,4,6-triiso­propyl­phen­yl)sulfon­yl]aziridine
Source: Acta Crystallogr E Crystallogr Commun. 2015 Jun 3;71(Pt 7):o438–9. doi: 10.1107/S2056989015010221 (PMC4518957; doi:10.1107/S2056989015010221)

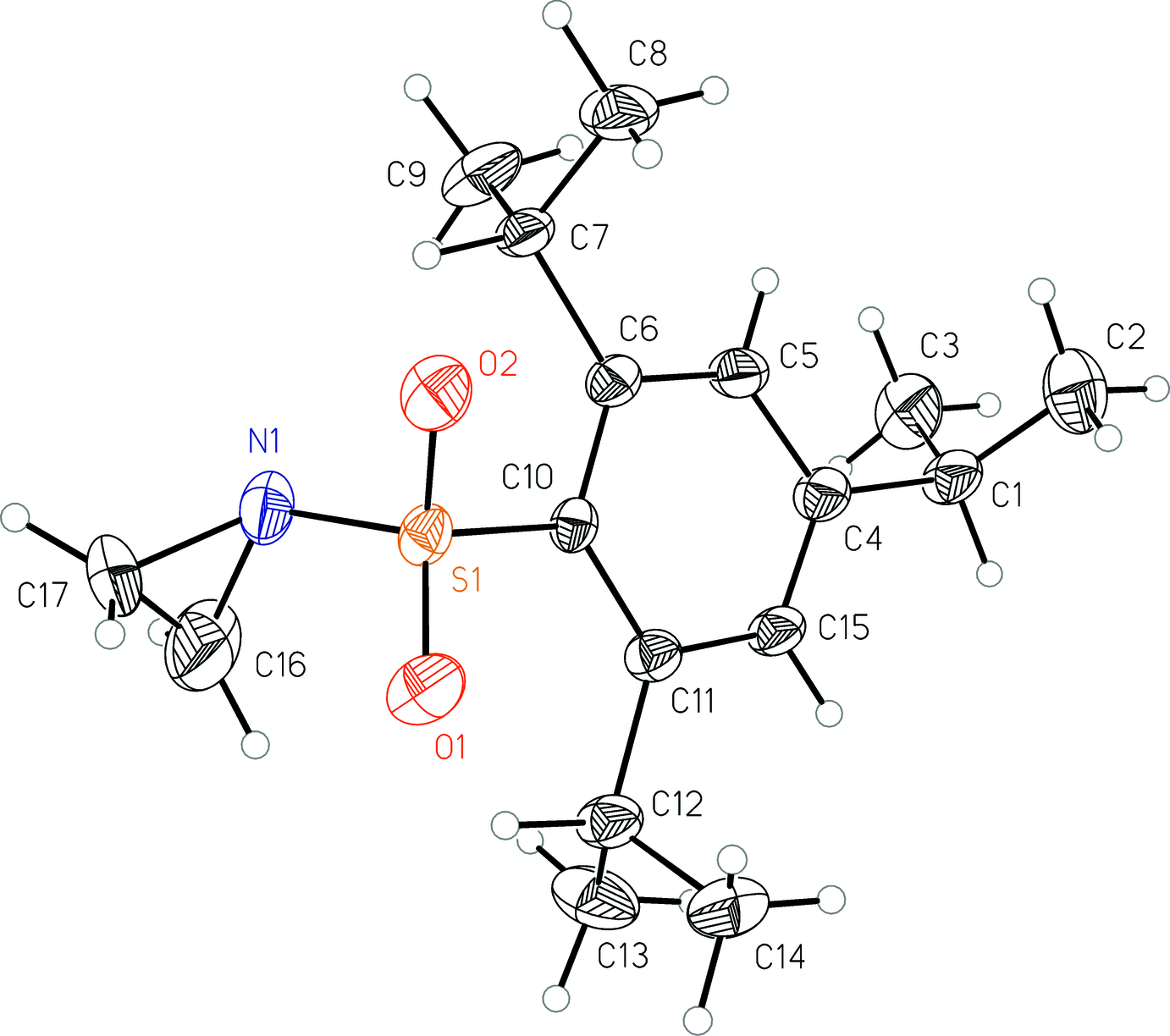

Supplement: Supplementary file 4 [file e-71-0o438-fig1.tif]
